# A Locomotor Innovation Enables Water-Land Transition in a Marine Fish

**DOI:** 10.1371/journal.pone.0011197

**Published:** 2010-06-18

**Authors:** Shi-Tong Tonia Hsieh

**Affiliations:** Department of Organismic and Evolutionary Biology, Harvard University, Cambridge, Massachusetts, United States of America; Ecole Normale Supérieure de Lyon, France

## Abstract

**Background:**

Morphological innovations that significantly enhance performance capacity may enable exploitation of new resources and invasion of new ecological niches. The invasion of land from the aquatic realm requires dramatic structural and physiological modifications to permit survival in a gravity-dominated, aerial environment. Most fishes are obligatorily aquatic, with amphibious fishes typically making slow-moving and short forays on to land.

**Methodology/Principal Findings:**

Here I describe the behaviors and movements of a little known marine fish that moves extraordinarily rapidly on land. I found that the Pacific leaping blenny, *Alticus arnoldorum*, employs a tail-twisting movement on land, previously unreported in fishes. Focal point behavioral observations of *Alticus* show that they have largely abandoned the marine realm, feed and reproduce on land, and even defend terrestrial territories. Comparisons of these blennies' terrestrial kinematic and kinetic (i.e., force) measurements with those of less terrestrial sister genera show *A. arnoldorum* move with greater stability and locomotor control, and can move away more rapidly from impending threats.

**Conclusions/Significance:**

My results demonstrate that axial tail twisting serves as a key innovation enabling invasion of a novel marine niche. This paper highlights the potential of using this system to address general evolutionary questions about water-land transitions and niche invasions.

## Introduction

Concepts of key innovation fall into two main categories: 1) the causal role it plays in diversification [e.g. 1], [2]–[7]; and 2) how it promotes ecological opportunity [e.g. 5], [7], [8], [9]. The integrated pharyngeal jaw apparatus in cichlid fishes is a classic example of key innovation enabling rapid species diversification and the invasion and colonization of broad adaptive zones in lacustrine environments [5]. Most cases of key innovation such as feathers in birds [10], the turtle carapace [2], [3], and the snapping claw in alpheid shrimps [11], involve extreme morphological modifications that subsequently improve performance capacity [12]. The far fewer examples of subtle novelties (e.g. directional asymmetry in the feeding apparatus of snail-eating snakes [9] or the stabilized jaw articulation in New World jays to withstand cracking acorns, is likely due to the difficulty of their identification and discovery.

Moving from an aquatic to a terrestrial niche is challenging due to the dramatically different demands each environment places on the physiology and structure of an organism; yet this transition was a necessary step in tetrapod evolution. Moving on to land requires numerous innovations [13] to accommodate respiratory, structural, and locomotor challenges absent in a buoyant, aqueous environment. While there are numerous paleontological examples of morphological innovations enabling a major ecological transition [e.g.], [ 13], [14,15], their value is limited to inferences based only on preserved, hard structures. Detailed examination of extant organisms would thus facilitate a better understanding for the selective pressures and challenges associated with major niche or habitat shifts.

The highly-speciose ray-finned fishes (Actinopterygii), comprise more than 25,000 species. Although fishes ancestrally are obligatorily aquatic, air-breathing capabilities and amphibious behaviors are surprisingly widespread in this group [16]. On land, amphibious fishes typically are from freshwater environments and tend to move very slowly [17]–[21]; yet two (of 53) genera of comb-toothed blennies (Blenniidae) regularly move about with great agility and rapidity on land [22]. Found above the waterline in the intertidal zone, they have been the focus of respiratory [22], [23] and behavioral studies [24], [25]. These fishes feed on algae they scrape off of rocks above the water line, migrate with tidal patterns [25], [26], and even reproduce on land [25], [27]. The Pacific leaping blenny, *Alticus arnoldorum*, has such terrestrial habits that it actively defends a terrestrial territory, and will retreat into moist burrows in the rocks when the tide recedes, to await its return. If threatened on land, these blennies slip into crevices above the water line or porpoise across the water surface to other rocky outcrops, and have never been observed to escape into the water.

**Figure 1:**
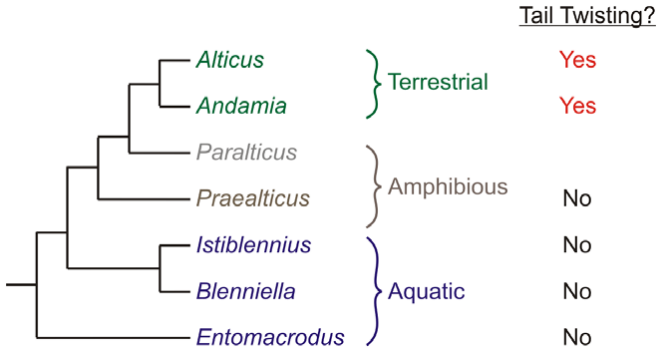
Tail twisting capability and ecological groupings of blenny genera examined in this study. Field observations showed that *Alticus* and *Andamia* are on land during both low and high tide. In contrast, *Paralticus* [35] and *Praealticus* exit the water infrequently during low tide (*pers. observ.*). *Istiblennius, Blenniella*, and *Entomacrodus* all are fully aquatic in their habits and only periodically emerge from water, despite being capable of breathing air. Tail twisting behavior in *Paralticus* is unknown because individuals were not available for examination. The phylogeny used here was obtained from Spring and Williams [28].

Six of the most closely-related genera to *Alticus* [28] exhibit different degrees of amphibious behavior. For ease of analysis, I have divided the genera into ‘aquatic,’ ‘amphibious,’ or ‘terrestrial’ groups based on field observations (Fig. 1). Although all of these genera can breathe air, aquatic blennies are seldom found out of water. When on land, they remain immobile until submerged by the next wave, or will flip about, seemingly randomly, until they return to water. Amphibious blennies can be periodically found feeding on land during low tide, close to the water line. However, they readily return to the water when disturbed and only make short forays on to land. The terrestrial blennies are extremely active on land, seldom submerge themselves under water, and move about actively dodging waves, feeding, and defending territories.

The goal of this study was to determine how two genera of ancestrally marine fishes have adapted to a terrestrial lifestyle. Initial high-speed videos showed that the terrestrial blennies perform an unusual axial tail twisting movement, not previously observed in any other known fish. By examining the kinematics and kinetics of terrestrial locomotion in these blennies and their non-terrestrial sister genera, I tested the hypotheses that this tail twisting motion (1) is uniquely derived in the terrestrial blennies; and (2) has facilitated their invasion of a terrestrial niche by conferring greater jump performance in comparison to their amphibious and aquatic sister genera. Results from comparative kinematics of terrestrial jumps advance a theory for how tail twisting evolved. Furthermore, these results present *Alticus* as a potential living model for understanding structural and functional challenges associated with a major environmental transition.

## Methods

### Animals

Blenny species examined in this study include *Alticus arnoldorum*, *Andamia reyi*, *An. tetradactyla*, *Blenniella caudolineata*, *Entomacrodus niuafooensis*, *E. striatus, Istiblennius lineatus*, and *Praealticus labrovittatus*. Only *Andamia spp*. were collected in Taiwan. All of the remaining species are from Guam. Data for *Entomacrodus* and *Andamia* were pooled within each genus because I was interested in differences among the blennies at the generic level. Intraspecific locomotor performance comparisons and quantification of locomotor kinematics were conducted in field marine laboratories in Guam (University of Guam Marine Lab) and Taiwan (Sun Yet Sen University, Kenting National Park), and at Harvard University (Cambridge, Massachusetts, USA). All experiments for this study were approved by the Institutional Animal Care and Use Committee at Harvard University. All animal collection was conducted in accordance to permit no. C00-008-04 issued by the Department of Agriculture in Guam and a permit issued by the Taiwan Council of Agriculture.

For the experiments at Harvard University (i.e., detailed kinematic and kinetics measurements), twenty live individuals each of *Alticus arnoldorum*, *P. labrovittatus*, and *B. gibbifrons* were shipped back to the laboratory. None of the other species were imported because their conditions rapidly deteriorate in captivity, or permits for live import were unavailable (i.e., *Andamia spp.* from Taiwan). In the lab, blennies were housed in groups of three in plastic shoeboxes modified to accommodate an airstone, a water inlet, and drain. Boxes were fit with a drain opposite the inlet to ensure cross-aquarium water flow. Fishes were fed twice daily with a spirulina and marine flake fish food mix. Full spectrum lights were set on a 12 hour light-dark cycle.

All locomotor trials were filmed at 250–1000 fps using one or two high speed video cameras.

### *Alticus* Terrestrial Kinematics

Since *Alticus* exhibited the most extreme terrestrial behavior, detailed description of this species' movements on land were completed. Kinematics of *Alticus* locomotor modes were quantified in three dimensions as individuals were induced to climb a vertical piece of Plexiglas and hop or jump along a horizontal surface. Each locomotor bout was divided into three phases based on body position and kinematics (see Results for a description of phases). Fish body midlines were manually digitized, then reconstructed into three-dimensional coordinates using custom software in MATLAB (Mathworks, Inc., USA). A fixed point on the body was used for locomotor velocity calculations.

To determine whether *Alticus* moved on land using distinct locomotor modes, 11 kinematic variables were quantified. These variables included take-off angle, duration of each locomotor phase as a percent of total bout duration, maximum body velocity (i.e., “maximum velocity”), and average three-dimensional body velocity (i.e., “average velocity”) and curling velocity for each phase. Take-off angle was measured as the angle formed by a straight line connecting the snout tip to the base of the tail and the horizontal plane. Curling velocity was defined as the speed with which the tail and snout moved with respect to one another. Positive velocities indicated that they were moving away from each other, representing speed of body extension. Linear regressions of variables against body length yielded no size effects. All variables were log-transformed before statistical analyses to satisfy assumptions of normality.

A principal components analysis (PCA) was first run to reduce the dimensionality of the data and to determine which variables were responsible for the greatest amount of variance in the data. A 90% trace criterion [29] was used to select the principal components (PC) to be used for further analyses. Individual variable loadings (i.e., the eigenvalues) and scatterplots of the principal components (PC) facilitated interpretation of PCA results.

The selected PCs were then analyzed with a descriptive discriminatory analysis (DDA) to examine categorical separations [e.g.], [ 29], [30,31]. Examination of a linear discriminant factor (LDF) plot and results of an ANOVA/Tukey-HSD comparison established whether group differences were described in one or two dimensions.

### Comparative Jumping Performance and Kinematics

All jump behaviors were recorded within two days of capture in Taiwan and Guam. For each jump, qualitative parameters (i.e., presence or absence of tail twisting and slipping) were recorded. Jumps were pooled according to blenny ecotype (i.e., terrestrial, amphibious, or aquatic). A total of 156 jump trials (i.e., 59 terrestrial, 49 amphibious, and 48 aquatic) were included in the final analysis. Differences in jump stability (i.e., slipping frequency) among ecotypes were assessed with a contingency analysis, followed by a Pearson's Chi-squared test.

### Comparative Jump Kinetics

Comparative jump force production among *A. arnoldorum, P. labrovittatus*, and *B. gibbifrons* – representing a terrestrial, amphibious, and aquatic blenny – was assessed using a novel, tri-axial optical force plate designed for these experiments [32]. Analog force plate response data were sampled at 1000 Hz and converted to digital form using an analog-digital converter (ADInstruments, Colorado Springs, CO, USA). All force data were filtered with a ninth-order, low-pass Butterworth filter set at a 20 Hz cut-off frequency, in the forward and reverse directions to eliminate filter-introduced time shifts. Force axes were assigned according to the right-hand rule: +X pointed opposite the direction of motion (aft), +Y pointed to the left of the jump, and +Z pointed down. Figure S1 shows a representative, filtered force trace from a jump by the terrestrial blenny, *Alticus arnoldorum*. All trials were also recorded with a high-speed camera and consumer grade camcorder (Sony Corporation) for general kinematics synchronization and quantification of jump distance. Activation of a trigger simultaneously terminated high-speed video filming and added a step change in electrical signal along a channel dedicated to recording trigger response.

A total of 43 trials were recorded from the terrestrial *A. arnoldorum* (10 individuals), 13 trials from the amphibious *P. labrovittatus* (2 individuals), and 12 trials from the aquatic *B. caudolineata* (3 individuals). Only those trials in which the blenny jumped from the center of the force plate, without touching the sides of the enclosure or the prod used to elicit the jump, were accepted for analysis. As a result of such stringent screening criteria, a highly-selective subset (21 total trials: aquatic = 5 jumps, amphibious = 4 jumps, terrestrial = 12 jumps) of the 68 recorded trials was selected for detailed analysis.

Multiple analyses of variance (MANOVA) tested the hypotheses that 1) forces produced during the preparatory phases were not statistically different among blenny ecotypes; and 2) terrestrial blennies generate greater propulsive force impulses than do amphibious and aquatic blennies. If significance was detected by a Pillai's Trace criterion, then an ANOVA and a *post-hoc* Tukey HSD test established pairwise differences.

### Statistics

All statistics were processed using JMP7.0.2 (SAS Institute, Inc., Cary, NC). Where applicable, data are presented as mean±S.E.M.

## Results

### Terrestrial locomotion is stereotyped

**Figure 2:**
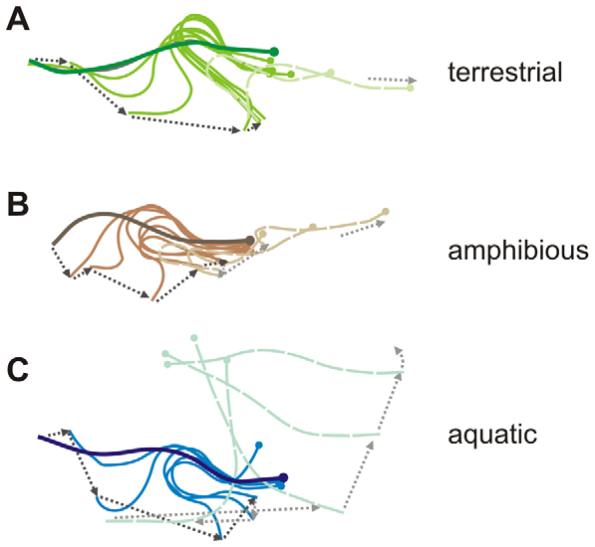
Dorsal midline splines taken from high-speed video of representative, stereotypical terrestrial jumps. Presented midlines include a **a**, terrestrial (*Andamia tetradactyla*), **b**, amphibious (*Praealticus labrovittatus*), and **c**, aquatic (*Blenniella gibbifrons*) blenny. The head is indicated by the filled circle, and the darkest midline corresponds to initial body position before movement. Lighter solid midlines indicate the body is contact with the ground, whereas the lightest dashed midlines indicate that the body is raised off the ground. Note the large yaw of the aquatic blenny, **c**, as its body leaves the surface. Dotted arrows indicate tail movement or overall body movement.

**Figure 3:**
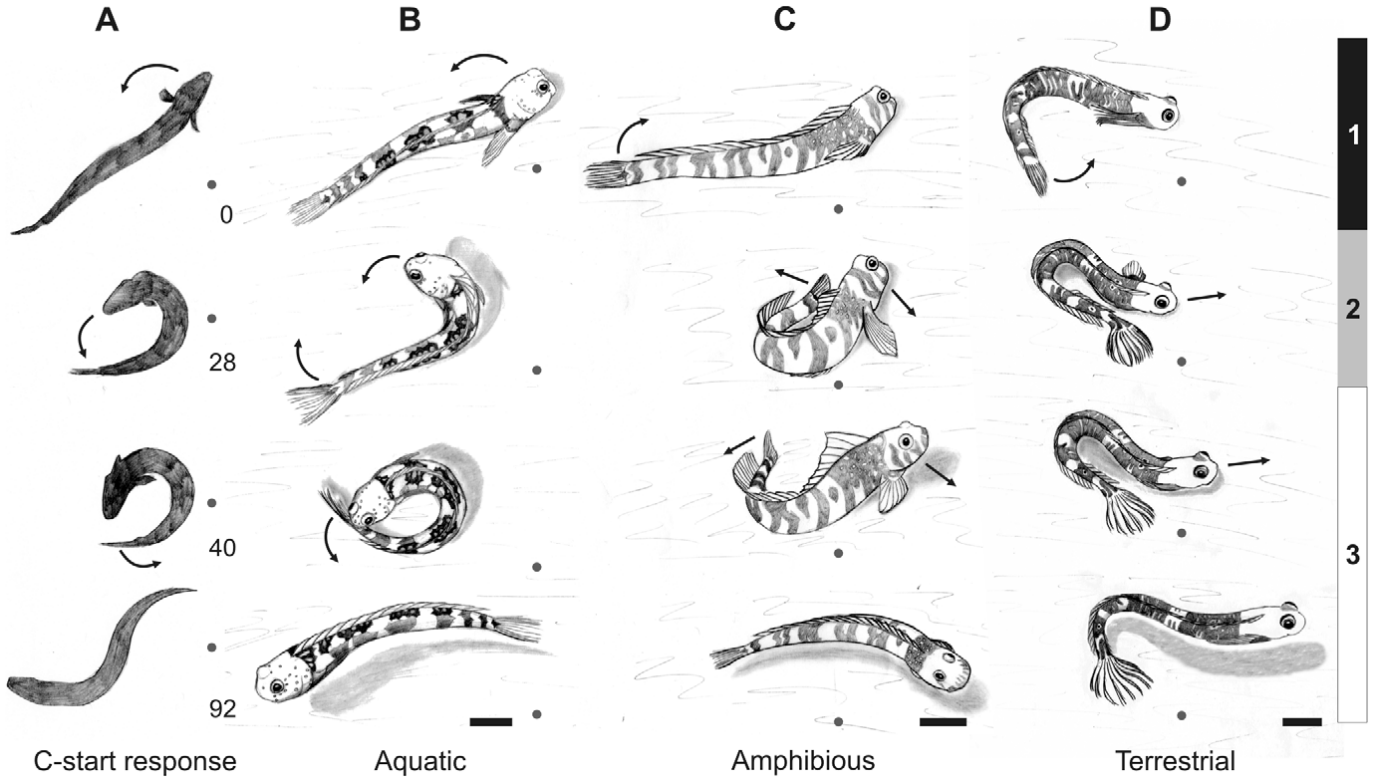
Comparison of aquatic escape and terrestrial jump maneuvers in aquatic, amphibious, and terrestrial fishes. **a**, An aquatic C-start escape response by a fully-aquatic fish, *Polypterus senegalensis*, modified from Tytell and Lauder [43]. **b**, An aquatic blenny (*Blenniella gibbifrons*) jumping on land. Note the lack of tail twisting and similar body position to the fish in panel **a**. **c**, An amphibious blenny (*Praealticus labrovittatus*) showing the stereotyped tail to head movement used for terrestrial locomotion. **d**, A terrestrial blenny, *Alticus arnoldorum*, demonstrating axial tail twisting. The numbered vertical bars to the right of panel **d** correspond to jump phases 1, 2, and 3, as shown in panels **c** and **d**. See text for descriptions of the phase kinematics. Blennies shown in panels **b–d** jumped off the same balsa wood surface. The terrestrial blenny never slipped, whereas all others did. Sketches were produced for greater visible clarity, and were traced from high-speed video frames. The gray circle serves as a fixed point. Five mm scale bars are provided at the bottom of panels **b–d**; panel **a** serves as a generic kinematic reference for a C-start escape response.

Although *Alticus* and *Andamia* exhibit the highest degrees of terrestriality, high speed video revealed that the aquatic, amphibious, and terrestrial blennies studied here all initiate terrestrial movement from a stereotypic posture: the tail is curled towards the head, forming a C-shape with the body, and then extended to push the body forward (Fig. 2). Tail movements in aquatic and amphibious blennies are limited to a side-to-side motion, like those of most other fishes (Fig. 3b, c and Movies S1 and S2). In contrast, terrestrial blennies twist their tail axially 90° before placement on the ground (Fig. 3d), using the lateral surface of their tail – rather than the ventral surface – to propel themselves forward (Movies S3, S4, S5). This unusual axial tail-twisting movement is unique to the two terrestrial genera (Fig. 1), and thus represents a kinematic innovation.

To facilitate kinematic comparisons, movements were divided into three phases, based on body position and kinematics (Fig. 3d). Phases 1 and 2 comprised the preparatory phases, whereas phase 3 comprised the propulsive phase. During phase 1, the tail is curled towards the head. The pectoral fin on the ipsilateral (i.e., concave) side of the body is folded against the body, whereas the contralateral pectoral fin remains extended. There was no detectable left-right preference for tail curling direction. Phase 2 is characterized by a pause in forward movement, during which the blenny maintains the U-shaped tail-to-head position, (Fig. S2, solid line). During this phase, terrestrial blennies twist their tail, pressing its lateral surface against the substrate and spreading the caudal fin rays. There may also be some slight body movement (e.g. rolling or shifting) as the fish prepares for the next phase. Phase 3 is the propulsive phase, starting with the first forward movement of the body, and ending when the tail loses contact with the surface.

### Distinct terrestrial locomotor modes in *Alticus*

**Figure 4:**
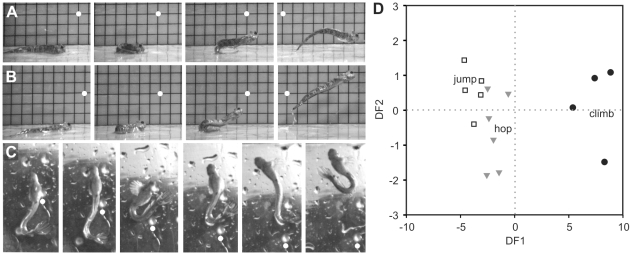
Three terrestrial locomotor modes performed by *Alticus arnoldorum*. **a**, Hopping, **b**, jumping, and **c**, climbing. **d**, Results of a discriminatory analysis on four principal components derived from two principal components analysis (PCA) models of preparatory and propulsive kinematics. All three locomotor modes are distinct (Wilks' Lambda: P<0.0001; ANOVA: F = 159.37, P<0.0001) and categorized with 93.7% accuracy (14 of 15 correct). See Tables 1 and S1 for a list of variables included in the PCA models.

*Alticus arnoldorum* was the most terrestrial blenny examined in this study. Hopping (Fig. 4a; Movie S3), jumping (Fig. 4b; Movie S4), and climbing (Fig. 4c; Movie S5), are distinct with respect to the speed with which they are performed (Table 1; ANOVA: F = 56.16, P<0.0001). Climbing and hopping are the two slowest locomotor modes used when foraging. On average, these blennies achieved a peak velocity of 0.38±0.06 m/s while climbing (range: 0.25–0.53 m/s), and 1.08±0.06 m/s while hopping (range: 0.93–1.29 m/s). Although hopping is similar to jumping in its positional characteristics, jumping is used for escaping threats and when fighting while defending territories, representing a much faster locomotor mode (peak velocity: 1.58±0.08 m/s; range: 1.39–1.87 m/s).

**Table 1:** Select kinematic variables characterizing climbs, hops, and jumps in the Pacific leaping blenny (*Alticus arnoldorum*).

Variable	Phase	Units	Climb (N = 4)	Hop (N = 6)	Jump (N = 5)
**Phase Duration**	1	%	47.6±3.4	58.9±4.4	54.7±3.5
	2	%	10.6±4.4	15.1±3.2	21.5±3.2
	3	%	41.8±3.1*†	26.0±2.1*	23.8±2.4†
**Mean Curl Velocity**	1	m/s (L/s)	−0.18±0.02 (−3.36±0.46)	−0.23±0.05 (−4.00±0.88)	−0.32±0.07 (−5.56±1.27)
	2	m/s (L/s)	0.05±0.01 (0.98±0.24)	0.07±0.02 (1.16±0.27)	0.08±0.02 (1.33±0.36)
	3	m/s (L/s)	0.18±0.04 (3.45±0.68)*†	0.55±0.05 (9.71±0.91)*	0.75±0.07† (12.96±1.12)
**Mean Body Velocity**	1	m/s (L/s)	−0.18±0.02 (−3.36±0.46)	−0.23±0.05 (−4.00±0.88)	−0.32±0.07 (−5.56±1.27)
	2	m/s (L/s)	0.05±0.01 (0.98±0.24)	0.07±0.02 (1.16±0.27)	0.08±0.02 (1.33±0.36)
	3	m/s (L/s)	0.18±0.04 (3.45±0.68)*†	0.55±0.05 (9.71±0.91)*	0.75±0.07 (12.96±1.12)†
**Take-off Velocity**		m/s (L/s)	0.06±0.08 (1.18±0.64)*	0.99±0.07 (17.31±1.33)*	1.46±0.07 (25.17±1.37)*
**Take-off Angle**		degrees	22.27±10.95	23.26±8.94	50.92±9.79

L/s: Lengths per second. Data are presented as mean±S.E.M.

*†Values marked with the same symbol indicate a significant difference between/among locomotor modes.

Two principal components analysis (PCA) models were developed for the preparatory and propulsive phase measures. The kinematic variables used in these models are presented in Table 1. Six phase 1 and 2 variables were included in the preparatory PCA model and the remaining five variables were included in the propulsive PCA model (Table S1). Using the 90% trace criterion [29], four principal components (PCs) for the preparatory model (96.73% variance) and two PCs for the propulsive model (93.88% variance) were selected for further analysis. The results of an ANOVA and Tukey HSD on each of the PC scores, and their kinematic interpretations are shown in Table 2.

**Table 2:** Principal components (PC) interpretations and means for each locomotor mode for preparatory and propulsive PCA models.

Phase	PC	Feature	Climb	Hop	Jump	*F*-ratio	*p*-value
Preparatory	PC1	Body velocities	−0.66±0.71	−0.30±0.58	0.89±0.63	1.58	0.2468
	PC2	Phase 1 duration and curling velocity	−0.95±0.55	0.26±0.45	0.45±0.49	2.09	0.1668
	PC3	*Interpretation unclear*	0.29±0.46	0.02±0.38	−0.23±0.41	0.33	0.7243
	PC4	Duration of Phase 2 pause in body movement	−0.59±0.33	0.27±0.27	0.15±0.29	2.29	0.1435
Propulsive	PC1	Phase 3 velocities (body and curling)	−2.81±0.43*†	0.47±0.35*	1.68±0.39†	**31.76**	**<0.0001**
	PC2	Take-off body angle	0.52±0.38	−0.49±0.31	0.17±0.34	2.32	0.1411

Results of an ANOVA for significant differences among locomotor means along each principal component are shown in the final two columns.

*†Locomotor mode means with the same symbol are significantly different from each other (p<0.05), as determined by post-hoc Tukey-HSD pairwise comparisons.

Preparatory phase kinematics were indistinguishable among the three locomotor modes, supporting the descriptive observation above that the initial body movements are stereotyped, regardless of locomotor mode. In contrast, the propulsive PCA model separated jumping and hopping from climbing. PC1 for the propulsive phase model showed that jumping and hopping exhibited significantly greater Phase 3 velocities (i.e., body, curling, and maximum velocities) than climbing (Table 2). However, jumping and hopping had statistically similar means to each other.

A descriptive discriminatory analysis (DDA) determined whether a combination of the preparatory and propulsive PC scores could be used to discriminate among the three locomotor modes [e.g.], [ 29], [30,31]. Interestingly, the amount of variance explained by each of the PCs in their respective models did not dictate their importance in discriminating among the locomotor modes. A stepwise discrimination procedure revealed that a minimum of four PCs (preparatory phase PC2 and PC4, and propulsive phase PCs) were necessary to distinguish among the three locomotor modes with 93.7% accuracy (Wilks' Lambda: P<0.0001; ANOVA: F = 159.37, P<0.0001). Propulsive PCs were the most discriminatory, with PC1 (body linear and curling velocities), and PC2 (take-off body angle) having comparable discriminatory power (Table S2).

### Terrestrial blennies exhibit the greatest jump performance on land

As hypothesized, terrestrial blennies outperformed both amphibious and aquatic blennies when moving on land. Terrestrial blennies jumped significantly farther than aquatic blennies (terrestrial: 12.5±1.4 cm; aquatic: 1.8±0.2 cm; ANOVA: F = 6.010, P = 0.012), but not significantly farther than amphibious blennies (5.3±0.7 cm). A second proxy for jump performance, slipping rate, indicated that terrestrial blennies also performed superior to the other two ecotypes (contingency analysis and Pearson's Chi-squared: χ^2^ = 123.396; P<0.0001). Whereas aquatic blennies slipped in 87.5% of the trials (N = 48) and amphibious blennies slipped in 73.5% of the trials (N = 49), terrestrial blennies never slipped in 59 total trials.

### Comparative Jump Kinetics

Based on the kinematic results above, two specific hypotheses were tested regarding comparative jump kinetics: 1) forces produced during the preparatory phases (1 and 2) among terrestrial blennies are identical to those produced during the same phase for amphibious and aquatic blennies, reflecting the stereotyped kinematics of initial movements during jumping on land; and 2) terrestrial blennies generate greater propulsive force impulses (X and Z) than do amphibious and aquatic blennies during the propulsive phase 3, reflecting the greater jump distances. Forces produced in the medio-lateral directions (Y) do not contribute to overall jump distance, so were not included in the calculation of propulsive force impulses.

Results from a MANOVA on preparatory phase jump kinetics (X, Y, Z, and total force impulse during phases 1 and 2) yielded no significant differences among blenny ecotypes (Pillai's Trace: F = 1.53, P = 0.17), indicating that the locomotor mechanics during the preparatory phase are similar among all these blennies.

Comparisons of propulsive phase jump kinetics (X, Y, and Z force impulse during phase 3, and jump distance) yielded more complicated results, mostly supporting the second hypothesis. On average, terrestrial blennies produced greater fore-aft (X-axis) force impulses (4.67±0.36 mN⋅s) during phase 3 than both aquatic (2.25±0.6 mN⋅s) and amphibious blennies (2.12±0.49 mN⋅s; ANOVA: F = 10.57, P<0.0009). However, no significant differences were detected among the ecotypes for medio-lateral (Y; F = 0.71, P = 0.50) and vertical (Z; F = 1.03, P = 0.38) force production, despite the significantly greater jump performance by terrestrial blennies.

## Discussion

The intertidal zone is a particularly challenging environment in which to live due to unpredictable and powerful wave impact on exposed rock [33], [34]. Although blennies frequently inhabit the intertidal, most are aquatic and benthic in their habits [35]. The terrestrial blennies (*Alticus* and *Andamia*) studied here have taken terrestriality to an extreme, in spite of extremely demanding conditions. Terrestrial blennies perform many essential behaviors on land, including feeding and reproducing, and they even defend terrestrial territories. Observations in the field showed that *Andamia tetradactyla* and *A. reyi* sleep at night in rock depressions above the water line but within the splash zone. When periodically wetted by spray from waves, *Alticus sp.* is reported to remain indefinitely out of the water [24]; pers. observ.], breathing through highly-vascularized skin [22]. This study shows that terrestrial blennies exhibit a key kinematic innovation that likely facilitated this major ecological transition. Their unique ability to twist their tail axially allows them to place the broad, lateral tail surface against the ground for propulsion, increasing their jump distance while also improving traction on frequently slippery, algae-covered rock surfaces. I present below some general observations of their territorial behavior, to emphasize the necessity of a mechanism for rapid and effective movements on land.

### Terrestrial blennies are highly territorial

Territories consisted of rock faces containing numerous short tunnels (usually less than 3 cm depth), holding a shallow volume of fluid accumulated during high tide. Whereas non-territorial blennies regularly moved over the entire rock face during high tide and disappeared during low tide, presumably migrating with the tide cycle, territorial individuals remained inside an exposed burrow during low tide. Blennies were not observed leaving the tunnels during low tide, suggesting the accumulated water within the tunnels is sufficient for keeping the fish moist – facilitating cutaneous respiration [24] – until the tide returned.

The highly terrestrial Pacific leaping blennies (*Alticus arnoldorum*) rely on rapid and acrobatic maneuvers to aggressively defend their terrestrial territories. Initial territorial displays consisted of rapid head-bobbing movements, resembling those displayed among lizards [36], with only the head exposed and the remainder of the body still within the burrow. Escalation of conflict led to emergence of the defender followed by tensing of the body, flaring of the dorsal, pectoral, and caudal fins, and lateral posturing towards the offender. Physical combat was common, rapid, and acrobatic, sometimes resulting in one or both of the blennies being knocked into the water. Blennies knocked off a rock immediately re-emerged on to land. None of the blennies were observed voluntarily entering the water during low or high tide.

### Greater performance on land among terrestrial blennies

The combination of living in the wave-swept intertidal and aggressive defense of terrestrial territories necessitates an effective means of moving about on land. Jumping is the fastest mode of locomotion measured for *Alticus*, most frequently used during territorial encounters and when escaping from impending threats (e.g., predators, waves, and aggressive conspecifics). It thus would be reasonable to expect terrestrial blennies to demonstrate kinematic or morphological specialization reflecting their more terrestrial lifestyle.

Qualitative kinematic comparisons yielded stereotyped kinematics of terrestrial movements on land, irrespective of ecotype. Among all ecotypes, movements were initiated by bringing the tail towards the head, curling their body into a C-shape then straightening the body to move forward. Using such stereotyped movements, detailed kinematic analyses showed that the terrestrial *Alticus* performed two to three distinct locomotor modes: hopping, jumping, and climbing. Hops and jumps were indistinguishable in both principal components models, but were differentiated in the discriminant analysis with 93.7% accuracy, suggesting that these two locomotor modes are distinct despite being very similar kinematically. It is nevertheless possible that jumping and hopping represent opposite extremes of a velocity continuum that is behaviorally modulated. While additional focal point studies are necessary to formalize when each locomotor mode is used, initial observations indicate that the diversity of locomotor modes facilitates slow, stable locomotion when feeding and fast, acrobatic locomotion when escaping large waves or competing for resources.

Comparisons among six closely-related genera showed that axial tail twisting is a kinematic innovation unique to the two terrestrial genera, and that these blennies are furthermore characterized by significantly greater jump performance on land. On average, terrestrial blennies jumped nearly seven times farther than aquatic blennies, and over twice as far as amphibious blennies. Greater jump performance in terrestrial blennies is likely due to the greater stability and traction afforded by planting the lateral aspect of the tail against the substrate, rather than the narrow, ventral surface. On identical substrata terrestrial blennies never slipped when jumping in comparison to both aquatic and amphibious blennies. Furthermore, if terrestrial blennies roll when airborne, they display a remarkable ability to correct body position, which permits landing upright and launching into the next jump almost immediately. Amphibious and aquatic blennies land on their side if they roll mid-air.

The kinetic data are consistent with greater jump performance among terrestrial blennies, showing that these blennies generated greater fore-aft force impulses, facilitating increased jump distance. In contrast, when considering their shorter jump distances, amphibious and aquatic blennies tended to generate proportionately greater vertical and medio-lateral force impulses. These forces contribute less to increasing jump distance [37], [38] and can be destabilizing [39], [40], by causing yawing and rolling about the center of mass.

### Evolutionary Implications

The stereotyped body movements when moving on land for all blenny genera studied here suggest a derivation from the C-start escape, an evasion maneuver common to most aquatic fishes [41]. A C-start is a reflexive, Mauthner cell-initiated response that involves curling the head towards the tail into a tight C-shape and rapidly straightening the body for propulsion [Fig. 3a [; 42], [43]]. When startled on land, the aquatic blenny kinematic response closely resembled an aquatic C-start (12 of 48 jumps; Fig. 3b); although the opposite kinematic sequence, in which the tail is curled towards the head, was also used. Amphibious blennies reacted similarly in response to a stimulus (Fig. 3c), but would also frequently roll their body onto the side of their tail to jump. This resulted in a similar tail position as that achieved by axial tail twisting in terrestrial blennies (Fig. 3d) without actually twisting the tail. Whereas terrestrial blennies always jumped farther and generated greater propulsive forces than aquatic blennies, amphibious blennies were intermediate to these two ecotypes. This jump performance ‘enhancement’ in terrestrial and amphibious blennies, as compared to aquatic blennies, supports the interpretation that pushing with the lateral surface of the tail promotes terrestrial locomotor performance.

Similar locomotor strategies, when employed both underwater and on land, may lead to a decrement in locomotor performance. For example, Gillis [20] showed that American eels utilize a lateral undulatory motion when swimming and when on land. The dramatic decrement in locomotor performance (i.e., lower velocity) on land was accompanied by significantly higher amplitude body undulations and higher-intensity electromyographic bursts in the axial musculature [44].

In contrast, whereas mudskippers (*Periophthalmus sp.*, Family Gobiidae or “gobies”) rely on a combination of lateral undulations and pectoral fin locomotion underwater [19], they perform at least two distinct types of locomotion on land: crutching and jumping [17], [18]. Crutching involves using the pectoral fins and tail as a tripod, providing stabilization while planting the pectoral fins to lift the body off the ground to move forward [18]. While crutching tends to be slow, jumping is employed as a rapid, terrestrial escape response, remarkably similar to the terrestrial kinematics observed among the aquatic blennies studied here. When jumping, mudskippers simultaneously bring their head and tail together before rapidly extending the body to propel the fish away from a threat [19]. Timing characteristics of water *versus* terrestrial escapes indicate that the maneuvers performed underwater may be Mauthner-mediated, whereas those on land are not – suggesting the use of a novel motor pathway for land-based escape behaviors [17].

Key innovations afford enhanced performance [9], [45], [46], promoting ecological opportunity. These innovations are sometimes credited with subsequent species diversification [5], [47] and radiation into new environmental niches [6], [8]. Although the terrestrial blennies studied here do not represent a case of dramatic species diversification, the large populations and widespread occurrence of these terrestrial blennies in the tropical Pacific Ocean is a testament to their remarkable success occupying a new niche on land. The axial tail twisting behavior serves as a kinematic innovation in this group of fishes [6], occurring simultaneous with a level of stability and maneuverability when moving on the intertidal coastline that other known fishes have yet to achieve. Initial preliminary analyses of axial skeleton (pers. observ.) and myosepta morphology (pers. comm., S. Gemballa) have yielded no obvious morphological modification enabling tail twisting. Yet, the kinematic trends of jump behavior among non-terrestrial and terrestrial blennies suggest potential morphological or neuromuscular modifications, warranting further study. The similarity in the preparatory curling motions in genera distributed among two largely aquatic fish families (i.e., blennies and gobies) suggests that these basic terrestrial movements have an ancestral origin preceding amphibious behavior, and may be more broadly observed among other amphibious genera. It furthermore suggests a case of convergent evolution of motor patterns, facilitating terrestrial locomotion. Active axial tail twisting remains unreported in any other genus of which I am aware. Therefore, future studies comparing tail use and muscle function during aquatic and terrestrial locomotion in the terrestrial blennies may provide greater insight into whether axial tail twisting is an adaptation for land locomotion, or if it was co-opted from another aquatic function. This lineage of fishes thus provides a unique glimpse of an evolutionary pathway by which a group of aquatic vertebrates has moved on to land, serving also as an appropriate system for understanding the evolution of locomotor control mechanisms enabling effective locomotion in two distinct environments.

## Supporting Information

Figure S1 A representative force trace recorded from a jumping terrestrial blenny, *Alticus arnoldorum*. All force data were filtered with a ninth-order, low-pass Butterworth filter set at a 20 Hz cut-off frequency, in the forward and reverse directions to eliminate filter-introduced time shifts. Force axes were assigned according to the right-hand rule: +X pointed opposite the direction of motion (aft), +Y pointed to the left of the jump, and +Z pointed down.
(0.61 MB TIF)

Figure S2 Time-dependent plots quantifying the different locomotor modes on land for the terrestrial *Alticus arnoldorum*. Graphs show body displacement and velocity, and curling velocity from a representative a, jump, b, hop, and c, climb. Curl velocity (solid curve, see Materials and Methods for definition) and body velocity (circles) correspond to the left y-axis, whereas body displacement (dashed line) corresponds to the right y-axis. Positive curl velocity indicates body extension. Circles are spaced at 4 ms intervals. Arrows pointing at gray circles indicate the start of each locomotor phase (see Fig. 3d and text). The fourth arrow indicates ‘tail off’ (‘to’) when the tail loses contact with the locomotor surface.
(4.34 MB TIF)

Table S1 Eigenvector results for each of the principal components in the preparatory and propulsive kinematic models.
(0.05 MB DOC)

Table S2 Features selected by stepwise linear discriminant analysis.
(0.03 MB DOC)

Movie S1 A jump performed by an aquatic blenny, *Blenniella gibbifrons*, off a balsa wood surface. Note that axial tail twisting is absent in this species and that it yaws uncontrollably once airborne.
(0.98 MB MOV)

Movie S2 The amphibious blenny, *Praealticus labrovittatus*, performing a jump on a balsa wood surface. Note that it also curls the tail towards the head before propulsion; however, it does not twist its tail and therefore pushes off the substrate with the ventral tail surface.
(1.18 MB MOV)

Movie S3 Lateral view of hopping in the terrestrial blenny, *Alticus arnoldorum*. Video was filmed at 250 fps, covering approximately 550 ms of movement. Notice the stereotyped curling of the tail to the head before body extension and that the fish lands in position to immediately execute a subsequent hop.
(2.05 MB MOV)

Movie S4 Lateral view of jumping in the terrestrial blenny, *Alticus arnoldorum*. Video was filmed at 250 fps. The full movement takes place in approximately 600 ms. This locomotor mode is similar to a hop, but the body is extended at a much higher velocity. Blennies can jump over 3 body lengths in one leap.
(2.12 MB MOV)

Movie S5 Ventral view of a terrestrial blenny, *Alticus arnoldorum*, climbing up a vertical piece of Plexiglas. Video was filmed at 250 fps, and this sequence occurs within 700 ms. Plexiglas was used so that the attachment of the fish to the locomotor surface could be visualized. In their natural environment, fishes climbing up rocks do not slip as is seen here.
(2.44 MB MOV)
