# Supplementary figures and images for: A Locomotor Innovation Enables Water-Land Transition in a Marine Fish
Source: PLoS One. 2010 Jun 18;5(6):e11197. doi: 10.1371/journal.pone.0011197 (PMC2887833; doi:10.1371/journal.pone.0011197)

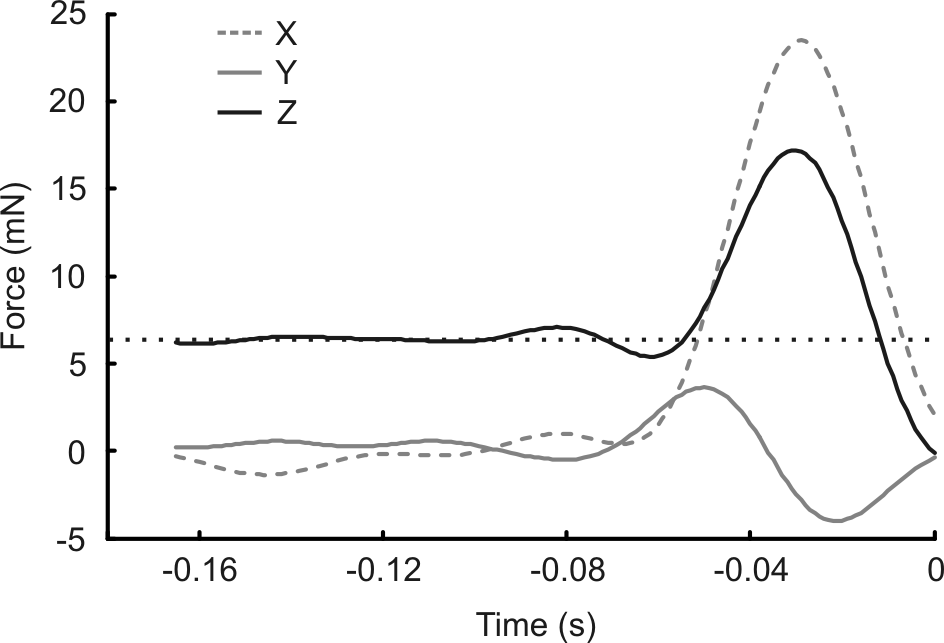

Supplement: Figure S1 — A representative force trace recorded from a jumping terrestrial blenny, Alticus arnoldorum. All force data were filtered with a ninth-order, low-pass Butterworth filter set at a 20 Hz cut-off frequency, in the forward and reverse directions to eliminate filter-introduced time shifts. Force axes were assigned according to the right-hand rule: +X pointed opposite the direction of motion (aft), +Y pointed to the left of the jump, and +Z pointed down. (0.61 MB TIF) [file pone.0011197.s001.tif]

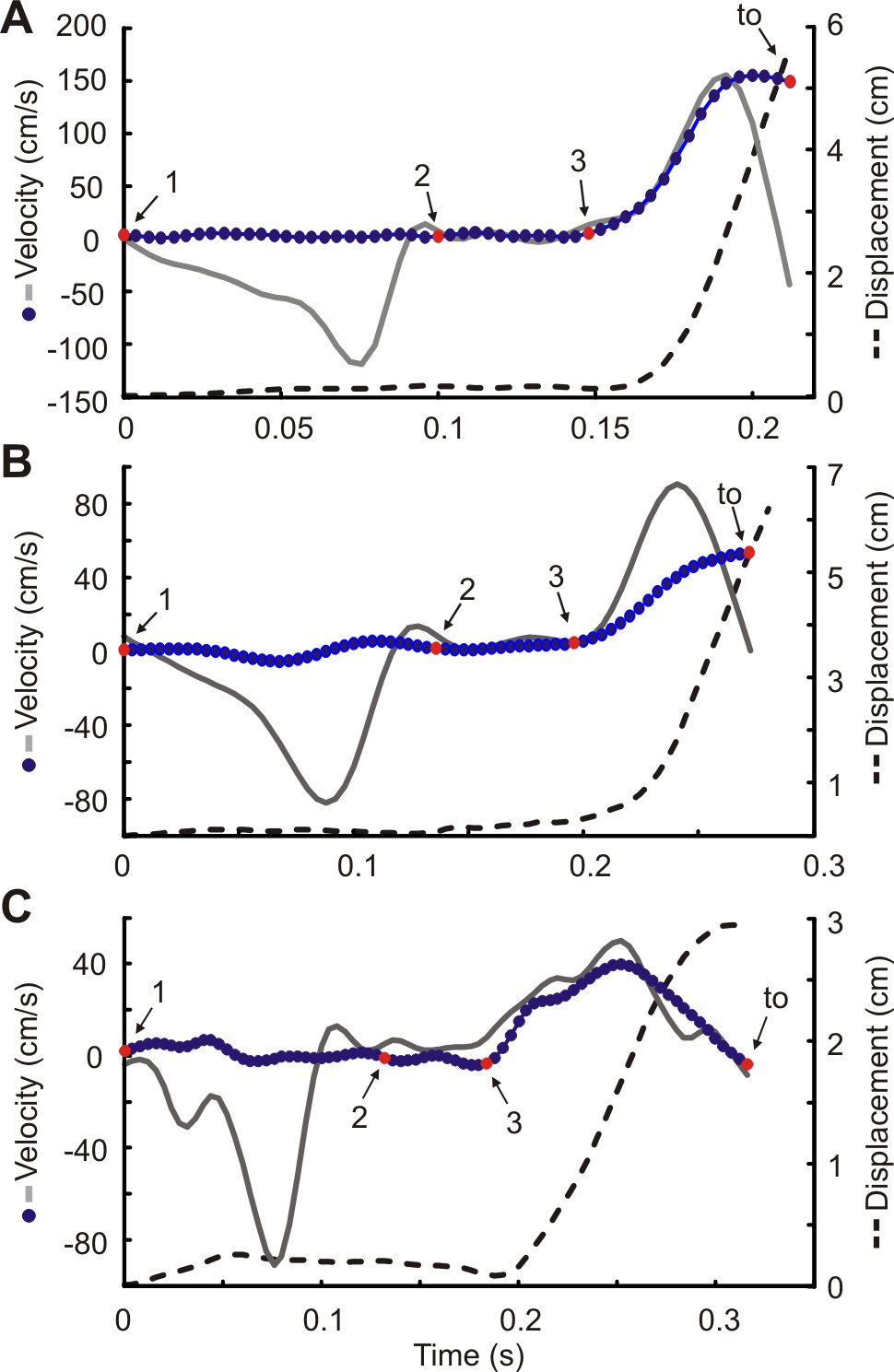

Supplement: Figure S2 — Time-dependent plots quantifying the different locomotor modes on land for the terrestrial Alticus arnoldorum. Graphs show body displacement and velocity, and curling velocity from a representative a, jump, b, hop, and c, climb. Curl velocity (solid curve, see Materials and Methods for definition) and body velocity (circles) correspond to the left y-axis, whereas body displacement (dashed line) corresponds to the right y-axis. Positive curl velocity indicates body extension. Circles are spaced at 4 ms intervals. Arrows pointing at gray circles indicate the start of each locomotor phase (see Fig. 3d and text). The fourth arrow indicates ‘tail off’ (‘to’) when the tail loses contact with the locomotor surface. (4.34 MB TIF) [file pone.0011197.s002.tif]
