# Supplementary material for: A Locomotor Innovation Enables Water-Land Transition in a Marine Fish
Source: PLoS One. 2010 Jun 18;5(6):e11197. doi: 10.1371/journal.pone.0011197 (PMC2887833; doi:10.1371/journal.pone.0011197)
Supplement: Table S1 — Eigenvector results for each of the principal components in the preparatory and propulsive kinematic models. (0.05 MB DOC) [file pone.0011197.s003.doc]

|  | Preparatory PC1 | Preparatory PC2 | Preparatory PC3 | Preparatory PC4 | Propulsive PC1 | Propulsive PC2 |
| --- | --- | --- | --- | --- | --- | --- |
| Phase 1 Body Velocity | 0.50 | 0.14 | -0.55 | -0.35 | -- | -- |
| Phase 2 Body Velocity | 0.49 | 0.39 | -0.06 | 0.08 | -- | -- |
| Phase 3 Body Velocity | -- | -- | -- | -- | **0.49** | -0.21 |
| Maximum Body Velocity | -- | -- | -- | -- | **0.48** | -0.20 |
| Phase 1 Curling Velocity | -0.38 | 0.52 | 0.23 | 0.13 | -- | -- |
| Phase 2 Curling Velocity | 0.41 | 0.18 | 0.77 | -0.36 | -- | -- |
| Phase 3 Curling Velocity | -- | -- | -- | -- | **0.49** | -0.05 |
| % Phase 1 Duration | -0.01 | 0.66 | -0.17 | 0.38 | -- | -- |
| % Phase 2 Duration | 0.45 | -0.31 | 0.16 | 0.76 | -- | -- |
| % Phase 3 Duration | -- | -- | -- | -- | **-0.44** | 0.18 |
| Take-off Angle | -- | -- | -- | -- | 0.32 | **0.94** |
| Eigenvalue | 2.37 | 1.98 | 0.76 | 0.51 | 4.03 | 0.67 |
| % Variance | 39.51 | 32.95 | 12.74 | 8.55 | 80.65 | 13.40 |
| Cumulative % | 39.51 | 72.46 | 85.19 | 93.74 | 80.65 | 94.05 |

All variables were log-transformed to satisfy assumptions of normality.
