# Supplementary material for: A Locomotor Innovation Enables Water-Land Transition in a Marine Fish
Source: PLoS One. 2010 Jun 18;5(6):e11197. doi: 10.1371/journal.pone.0011197 (PMC2887833; doi:10.1371/journal.pone.0011197)
Supplement: Table S2 — Features selected by stepwise linear discriminant analysis. (0.03 MB DOC) [file pone.0011197.s004.doc]

| PCs retained from stepwise procedure | First linear discriminant function coefficients^*^ | Rank of importance in linear group discrimination |
| --- | --- | --- |
| Propulsive PC1 | -2.86 | 1 |
| Propulsive PC2 | 2.35 | 2 |
| Preparatory PC4 | 1.63 | 3 |
| Preparatory PC2 | 0.81 | 4 |

* Only LDF1 is presented here because categories were significantly distributed only along this dimension. The absolute value of the coefficient in the discriminant function indicates its relative importance in classifying the categories.
